# Perinatal Asphyxia Affects Rat Auditory Processing: Implications for Auditory Perceptual Impairments in Neurodevelopmental Disorders

**DOI:** 10.1371/journal.pone.0015326

**Published:** 2010-12-23

**Authors:** Fabrizio Strata, Ivilin P. Stoianov, Etienne de Villers-Sidani, Ben Bonham, Tiziana Martone, Tal Kenet, Edward F. Chang, Vincenzo Vincenti, Michael M. Merzenich

**Affiliations:** 1 Department of Neuroscience, Section of Physiology, University of Parma, Parma, Italy; 2 Keck Center for Integrative Neuroscience, Coleman Memorial Laboratory, Department of Otolaryngology - Head and Neck Surgery, University of California San Francisco, San Francisco, California, United States of America; 3 Department of General Psychology, University of Padova, Padova, Italy; 4 Department of Neurology and Neurosurgery, Montreal Neurological Institute, McGill University, Montreal, Canada; 5 Epstein Laboratories, Department of Otolaryngology - Head and Neck Surgery, University of California San Francisco, San Francisco, California, United States of America; 6 Department of Clinical Physiopathology, Division of Otolaryngology, University of Torino, Torino, Italy; 7 Department of Neurology, Athinoula A. Martinos Center for Biomedical Imaging (MGH/Martinos), Massachusetts General Hospital, Charlestown, Massachusetts, United States of America; 8 Department of Neurosurgery, University of California San Francisco, San Francisco, California, United States of America; 9 Department of Otolaryngology, Head and Neck Surgery, University of Parma, Parma, Italy; Chiba University Center for Forensic Mental Health, Japan

## Abstract

Perinatal asphyxia, a naturally and commonly occurring risk factor in birthing, represents one of the major causes of neonatal encephalopathy with long term consequences for infants. Here, degraded spectral and temporal responses to sounds were recorded from neurons in the primary auditory cortex (A1) of adult rats exposed to asphyxia at birth. Response onset latencies and durations were increased. Response amplitudes were reduced. Tuning curves were broader. Degraded successive-stimulus masking inhibitory mechanisms were associated with a reduced capability of neurons to follow higher-rate repetitive stimuli. The architecture of peripheral inner ear sensory epithelium was preserved, suggesting that recorded abnormalities can be of central origin. Some implications of these findings for the genesis of language perception deficits or for impaired language expression recorded in developmental disorders, such as autism spectrum disorders, contributed to by perinatal asphyxia, are discussed.

## Introduction

Perinatal asphyxia (PA), a shortage of oxygen occurring before, during or after birth, has been linked to higher risk for neurodevelopmental disabilities, ranging along a broad continuum of severity. Significant sequelae of PA include death, cerebral palsy, mental retardation and untreatable seizure [1], [2], [3]. Epidemiological and clinical studies, however, have also recorded a correlation between PA and schizophrenia or autism [2], [4]. According to the hypothesis advanced by Simon [5], by damaging brainstem (BS) auditory nuclei, PA affects sound/speech perception resulting in poor language development, the most striking and consistent symptom in both moderately and severely developmentally impaired (e.g. autism spectrum disorders) infants. Damage to brain stem nuclei is a main pathological findings in infants who died by asphyxia [6] and disruption in the neuron morphology of these nuclei has been documented in autistic brains [7].

Experimental data from monkeys exposed to various patterns of hasty umbilical cord clamping at birth, showed a ranking order in brain structures' susceptibility to PA, with auditory BS nuclei found at the top of the rank order of brain structures that are susceptible to PA [8], [9], [10]. These monkeys showed also increased hearing threshold and reduced orientation to sounds, modality specific impairments similar to those reported in autistic infants [11], [12], [13], [14], [15]. Reduced sound detection capabilities, increased hearing threshold, degraded tonotopic representations, and reduced temporal precision at both subcortical (i.e. brainstem) and cortical levels have been recently reported in rats exposed to experimental PA [16]. It was suggested that degraded spectral and temporal sound features could underlie reduced perceptual capabilities of behaviorally significant complex sounds such as animal vocalizations or speech.

As part of a more extensive effort to clarify the sequelae of PA on auditory processing, the present study aimed at investigating phenomenology and mechanisms underlying the observed degraded spectral and temporal features of sound processing and their possible relation to auditory perception impairments that have been reported in autism spectrum disorders [14], [15], [17].

## Results

### Degraded tone-evoked responses in PA rats

**Figure 1:**
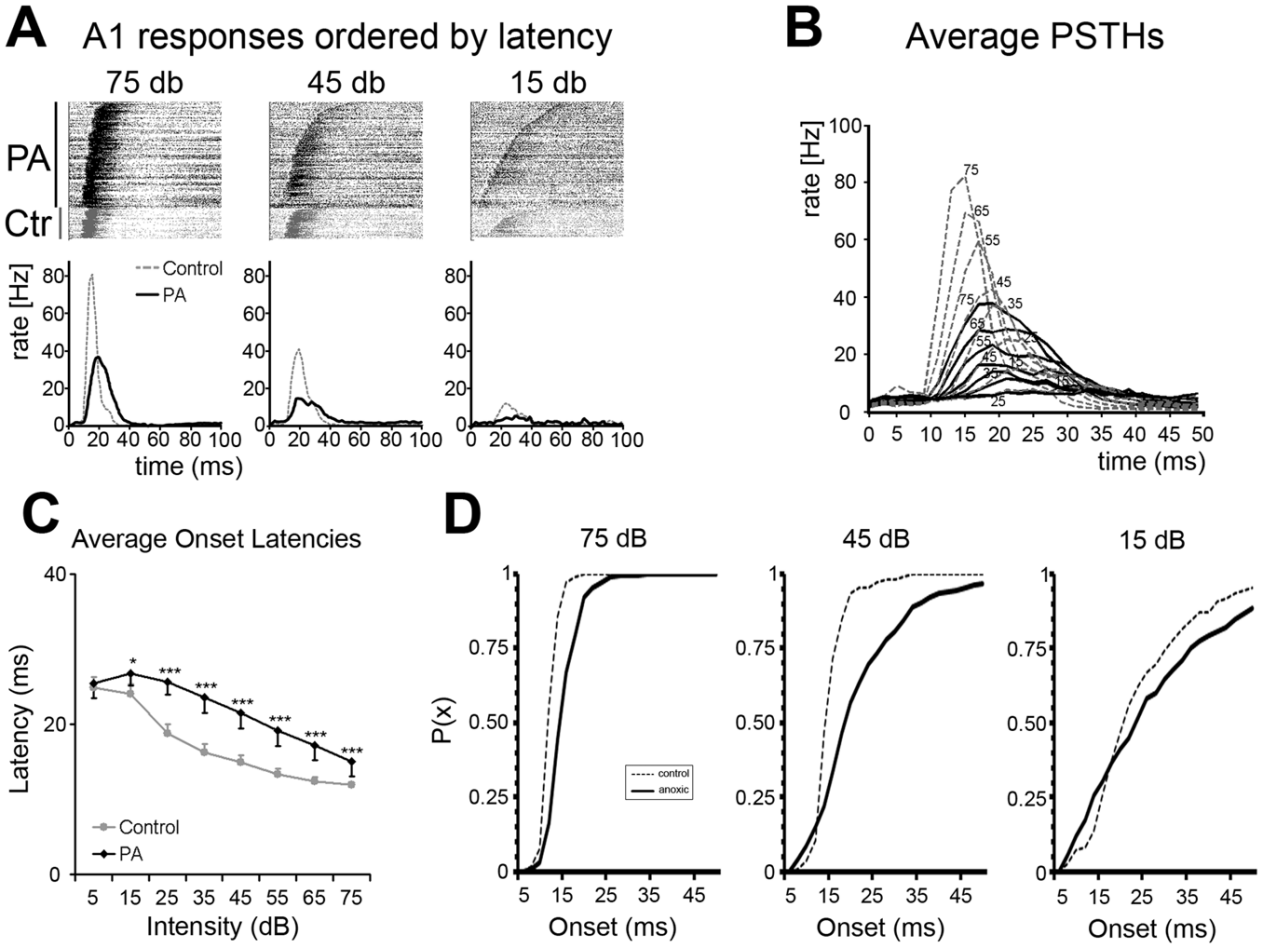
Tone evoked response latency differences in control and PA rats. (**A**) Panels showing the rasterized responses to tone pips for each recorded neuron in control (grey, bottom part) and PA rats (black, top part) at 3 test intensity levels: 75 (left) 45 (center) and 15 (right) dB. Each line corresponds to a single experiment. Below the raster plots their respective cumulative PSTHs derived from all responses. Grey represents controls and black PA. The same color code was used in all following illustrations. (**B**) Superimposed PSTHs derived from responses obtained for all intensity levels. Note amplitudes and peak latencies differences between controls and anoxics. (**C**) Plot summarizing response latencies calculated as average time in ms at PSTHs onsets shown in B. Response latencies decreased monotonically with increasing intensities in both groups. (**D**) Plots showing onset latency cumulative probability for three different intensities (75 dB left; 45 dB center and 15 dB right) in controls (dashed lines) and PA (solid line) rats. An increase in longer-latency response neurons in PA rats is highlighted by the rightward shift of their cumulative distribution. It is worth noting that, in PA rats, by decreasing the sound intensity there is an increase in shorter-latency responses. X-axis represents onset latency in ms; y-axis the probability for a given onset latency value.

Tuning curves (TCs) and peri-stimulus time histograms (PSTHs) were derived by recording tone-evoked responses from neurons within the middle cortical layers of the primary auditory cortex, A1, in adult normal (control) and experimental (exposed to PA) rats. As shown in **Fig. 1A–C** response onset latency is inversely correlated with stimulus intensity in both control and PA rats. Response onset latencies were shorter for higher-intensity tones lengthening progressively with lower stimulus intensities (**Fig. 1A–B**). In control rats, average onset latencies were 11.92±0.32 ms and 14.91±0.93 ms for 75 dB and 45 dB sound intensities respectively. Average onset latencies were longer in PA neurons, and they were 15.05±0.17 and 21.50±0.50 ms for 75 dB and 45 dB sounds respectively (**Fig. 1B–C**). It's worth stressing that, responses to 75 dB sounds in PA rats occurred with onset latencies overlapping those to 35–45 dB sounds in controls (**Fig. 1B–C**). Mixed ANOVA (within intensities and between groups) revealed main effect of intensity [F(7,490) = 65.2; p<0.001] and groups [F(1,490) = 98.1; p<0.001] and interaction between them [F(7,490) = 4.7; p<0.001]. It should also be noted that a main difference in A1 latency distribution was due to an increase in longer-latency neurons in PA vs. control rats, as shown by the rightward shift in the cumulative probability distribution of the onset vs. intensity plot (**Fig. 1D**). The difference was particularly evident for the 45 dB sounds. Finally, short-latency responses were still recorded at some A1 sites in PA rats.

### PA affects burst strength and duration but not inter-spike intervals

**Figure 2:**
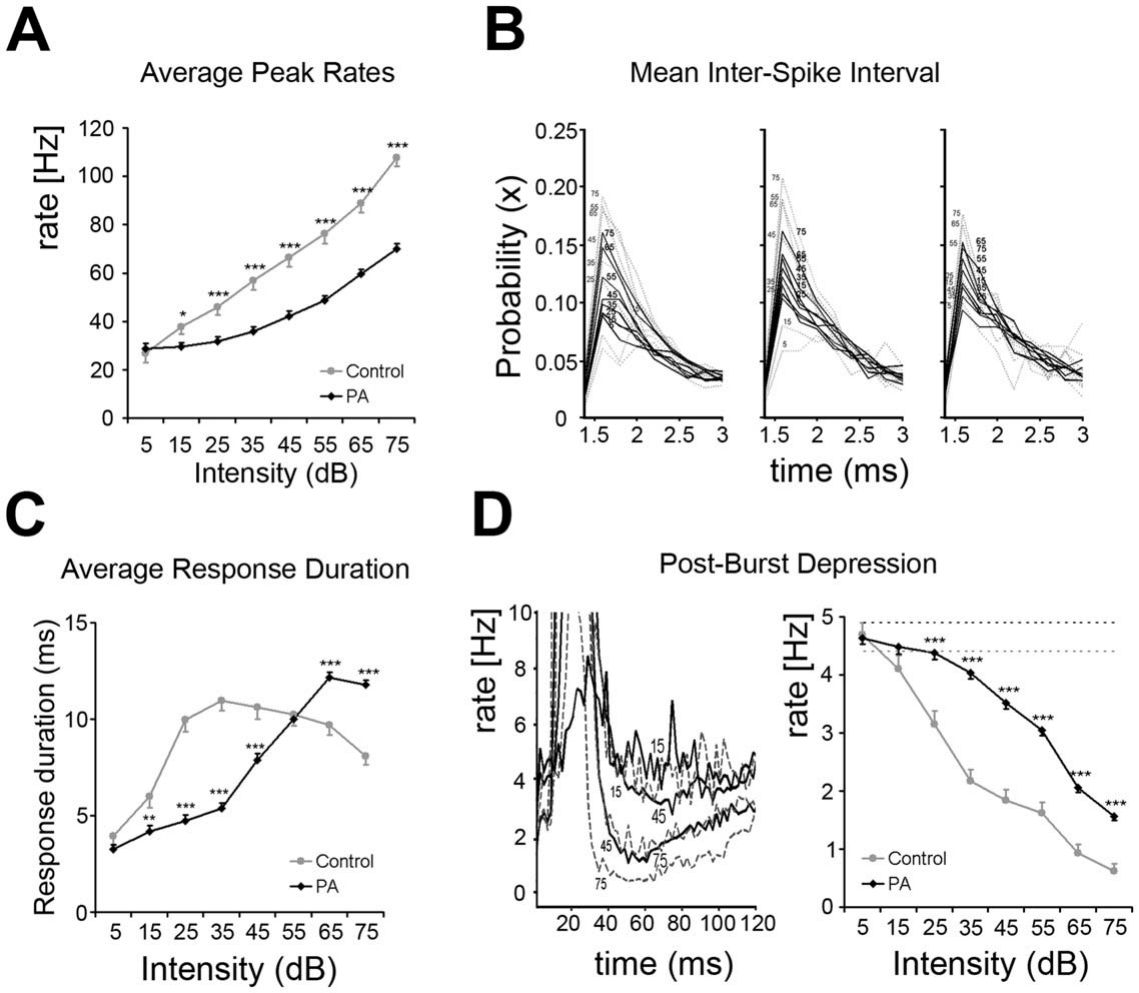
PA affects response strength and duration but not cortical bursting properties. (**A**) Plot summarizing response amplitudes to tone pips calculated as average spike rates at PSTHs peaks shown in **Fig. 1B**. Response amplitudes decreased monotonically with decreasing intensities for both groups. (**B**) Inter-spike intervals calculated between the 1^st^-2^nd^, 2^nd^-3^rd^, and 3^rd^-4^th^ spikes of the burst response. Note that mean ISIs did not differ between control (dashed grey lines) and PA (black solid lines) rats. In PA neurons the probability of detecting those ISIs was reduced at most intensities tested. Interestingly, the maximal probability was similar in all ISI examined. X-axis represents ISI values in ms, with a bin of 0.2 ms; y-axis the probability for a given ISI-bin-value at different intensities. (**C**) Plot summarizing response durations calculated as average time in ms at PSTHs onset-to-offset shown in **Fig. 1B**. Asterisks mark significant differences. (**D**) *Left panel*. Post-burst inhibition (PBI) recorded after excitatory responses illustrated for three different sound levels (75, 45 and 15 dB). Grey dashed lines represent controls; black solid lines represent data from PA rats. The magnitudes of PBI were a function of sound intensity and proportional to response amplitude; inhibition was the strongest when it followed high-intensity stimulation, decreasing monotonically with decreasing intensity. *Right panel*. PBI vs. intensity function. PBI was calculated as average firing rate in spike-per-sec across a post-firing window ranging between 10 and 50 ms after the excitatory response. Post-burst rates were below average spontaneous activity rates in both groups. Note the rightward shift in the PBI vs. intensity function in PA neurons, suggesting that PBI recorded from PA rats was sharply reduced.

Responses in control rats were robust for high-level sounds, and increased monotonically with increasing intensities (**Fig. 2A**). When calculated at the peak of the PSTH, average firing rates were 107.7±3.7 spikes/sec and 66.2±3.8 spikes/sec for responses to 75 dB and 45 dB tone levels respectively. Response strengths recorded from PA rats were significantly lower, and average firing rates were 70.1±2.0 spikes/sec and 42.2±2.0 spikes/sec for 75 dB and 45 dB sounds levels respectively (**Fig. 2A**, but see also **Fig. 1B**). Responses in PA neurons also grew monotonically as a function of sound intensity. In PA neurons response strengths to 75 dB tones roughly corresponded to those evoked by 45 dB tones in controls. Mixed ANOVA revealed significant differences within intensities [F(7,490) = 288.2; p<0.001], between groups [F(1,490) = 34.5; p<0.001] and interaction between intensities and groups [F(7,490) = 26.5; p<0.001].

A1 neurons usually respond at stimulus onset with short spike trains (bursts). Inter-spike intervals (ISIs) are critical for defining bursting properties in sensory circuitries. ISIs depend on both synaptic and intrinsic neuronal properties [18]. ISIs calculated between the 1^st^ and the 2^nd^, the 2^nd^ and the 3^rd^, and the 3^rd^ and the 4^th^ spike of the burst were similar in control and PA rats (**Fig. 2B**), suggesting that PA does not affect this aspect of the temporal coding (or processing) of cortical excitatory neuronal response properties.

Burst response durations varied as a non-monotonic function of stimulus intensity (**Fig. 2C**). In controls, average response duration was 8.09±0.47 ms for 75 dB tones, increased with falling intensity to 10.97±0.54 ms for 35 dB tones, and then decreasing again with lower tone levels (i.e. 6.00±0.6 ms for 15 dB tones). In adult PA rats response durations to high-intensity stimuli were significantly longer than in controls, and they were 11.78±0.25 ms and 12.16±0.27 ms for 75 and 65 dB tones respectively (**Fig. 2C**). In PA neurons, however, mean durations decreased more rapidly with lower sound intensities, as shown by the rightward shift of the duration-intensity curve: response durations were 7.89±0.33 ms for 45 dB stimuli and 4.19±0.31 ms for 15 dB. Observations at stimulus levels higher than 75 dB were not recorded, and it may be that response durations in PA neurons would have decreased for louder stimuli. Mixed ANOVA revealed significant differences within intensities [F(7,490) = 82.6; p<0.001], between groups [F(1,490) = 15.4; p<0.001] and interaction between them [F(7,490) = 33.9; p<0.001].

PSTH response bursts were followed by short periods of firing rate depression known to be attributable to inhibitory mechanisms with a major role in response timing control [19], [20]. Post-burst inhibitions (PBIs) were function of sound intensities, they were maximal after high-intensity stimuli and decreased monotonically with decreasing intensity (**Fig. 2D**, *left panel*). In control neurons mean PBI was 0.6±0.1 spike/sec after responses to 75 dB stimuli and 1.8±0.2 spike/sec after 45 dB sounds. PBI recorded from PA rats were strongly reduced, and they were 1.6±0.1 spike/sec and 3.5±0.1 spike/sec for 75 dB and 45 dB sounds levels respectively (**Fig. 2D**, *right panel*). Both were well below normal average spontaneous activity rates (4.4 and 4.9 spikes/sec in control and PA rats, respectively). Mixed ANOVA revealed main effect of intensities [F(7,490) = 158.0; p<0.001], groups [F(1,490) = 112.1; p<0.001] and interaction between them [F(7,490) = 10.2; p<0.001]. Again PBI after responses to 75 dB sounds were comparable to those recorded after 45 dB tones in controls, consistent with the 20–30 dB shift observed for excitatory responses, and with an actively sustained, tight relationship between excitation and inhibition [19], [20].

### PA alters the tuning curve response time-variant pattern

**Figure 3:**
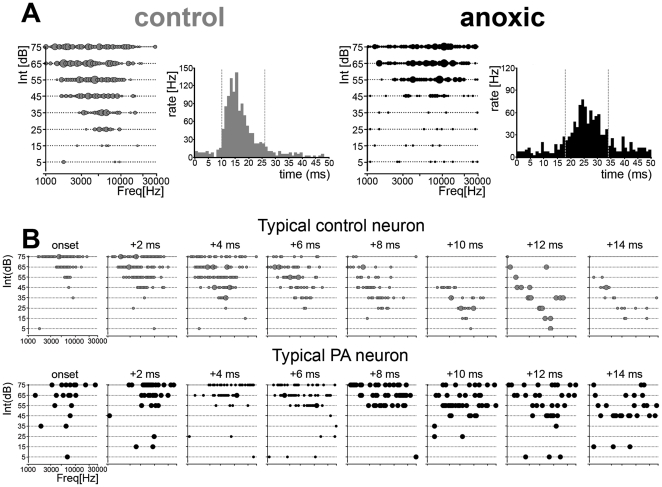
Tuning curves temporal dynamics. (**A**) Representative tuning curve examples obtained from one control (left) and one PA rat (right); PSTHs for those neurons are shown at the right of each example. Dashed lines mark the time windows selected for reconstructing response tuning dynamics based on a 2 ms bin. Note that response onset latency was increased in the sample PA neurons, while response strengths were reduced. (**B**) Temporal sequences of instantaneous tuning for single neurons recorded from one control (top) and one anoxic (bottom) rat. High-intensity tones corresponded to shorter latencies responses contributing to the PSTH's earlier phase; lower intensity tones corresponded to increasingly longer latency responses, contributing to the later phase of the PSTH. Time-slice per panel is 2 ms.

TCs are stationary representations of neurons' sensitivity in the intensity-frequency domain derived from accumulating simple responses based on PSTHs (**Fig. 3A**). To visualize TC dynamics, PSTH responses can be time-sliced in 2 ms bins, and instantaneous response selectivity (tuning) plotted as a function of time across the evoked onset-burst responses (**Fig. 3B**; see [21]). In such an analysis, neurons recorded from normal A1 (controls) first responded to a broad frequency range at higher stimulus intensity displaying reduced spectral selectivity (**Fig. 3B** *leftmost top panels*). A few ms later, evoked responses represented a narrower frequency range (higher spectral selectivity) at lower intensities, close to neurons' characteristic frequencies (CF; **Fig. 3B** *3 rightmost top panels*). Late diagonal bands of activity along low- and high-frequency borders, or V-shaped ‘caps’ of symmetric activity on TC low-intensity domains characterized PSTH's final time-slices, as exemplified in the two rightmost top panels of **Fig. 3B**. It is worth noting that control A1 neurons responded with higher spectral selectivity to lower-intensity tones when low-spectrally selective responses to higher-intensity sounds were mostly over. In adult PA rats, as shown in the lower record of **Fig. 3B**, this time-variant orderly pattern was lost. Responses to higher-intensity stimuli lasted longer than those recorded from control neurons (see also **Fig. 2C**), and persisted even during time-windows when responses to low-intensity stimuli were highest (**Fig. 3B** *rightmost lower panels*). Moreover, shorter response durations to low-intensity sounds made the ‘two events’ practically overlying.

### Degraded inhibitory processing in PA neurons

**Figure 4:**
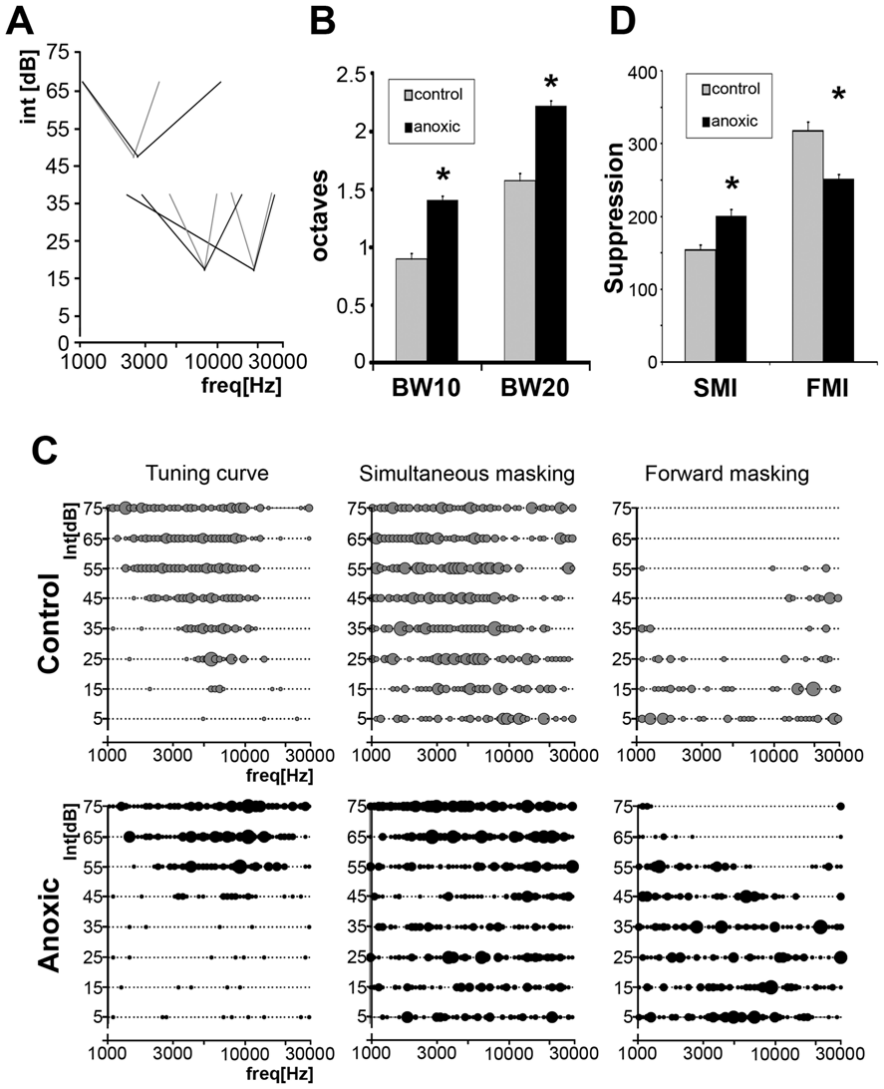
Altered inhibitory mechanisms in PA rats. (**A**) TC tips recorded for three best frequencies in control (grey) and PA (black) rats. The peak of each tip, derived from a single neurons sample, represents the CF or preferred frequency. Lines, connecting peaks and TC edges 20 dB above threshold, represent bandwidth of TCs 10 and 20 dB above each neuron's threshold (BW10 and BW20 respectively). TC bandwidths 10 and 20 dB above thresholds were significantly broader in PA rats. (**B**) Plot summarizing differences in bandwidth measured in octaves. A1 neuronal BW10 and BW20 were significantly broader in PA rats (asterisks; *U-test* p<0.05). (**C**) *Left panels* show representative examples of TCs obtained from one control (*Upper*) and one PA rat (*Lower*) with similar spectral features. *Center* and *right* show representative examples of simultaneous (*Center*) and forward (*Right*) masking inhibitions obtained with two-tone stimuli paradigms. (**D**) Plot summarizing the number of responses to probes inhibited by maskers played simultaneously (SMI) or preceding the probe (FMI). SMI was significantly increased in PA rats, that is, maskers randomly distributed across the intensity-frequency field suppressed a greater number of probe responses. FMI was instead significantly reduced in PA rats.

Spectral selectivity of A1 neurons was also reduced in mature PA rats. Sharpness of tuning was assessed by measuring TC bandwidths in octaves, 10 and 20 dB above neuron's threshold (BW10 and BW20 respectively). TCs recorded from PA rats' A1 neurons were significantly broader than those derived from controls (**Fig. 4A**). BW10 and BW 20 measured 0.90±0.05 and 1.57±0.06 octaves (mean±s.e.m.) in controls and 1.40±0.03 and 2.22±0.05 octaves in PA neurons, respectively (summarized in **Fig. 4B**).

Broader tuning and reduced post-burst depression were consistent with degraded inhibitory mechanisms [22], [23]. Abnormal inhibitory responses could be assessed by using two-tone presentations. For each pair a varying “masker” tone suppressed cortical responses to a fixed “probe” tone selected near neuron's CF, but had an intensity 10–20 dB SPL above threshold, in order to evoke reliable responses when presented alone. Maskers, presented in randomized manner, covered the whole tone pip intensity-frequency spectrum used to define a TC. Probe tone response suppression represented the masking inhibition. The two-tones were either presented simultaneously (simultaneous masking inhibition, SMI) or with an onset asynchrony of 40 ms (forward masking inhibition, FMI) [23], [24], [25].

Simultaneous two-tone stimulation in controls typically revealed one or two areas of response suppression around TC borders (**Fig. 4C**, *top center panel*). Probe responses were not suppressed when maskers failed to evoke neuronal responses. TCs with similar spectral features, shapes, CFs and BWs, were associated to very different sideband inhibitory areas (see also [23], [25]). Clear differences were observed in adult PA rats. SMI areas could be defined in less than one third of the cases (28%) and in some were not along TCs' borders but distant from them. In the other cases restricted SMI areas, or lack of them, were associated with a large number of probe sites at which response suppression was recorded almost randomly, broadly distributed all across the frequency-intensity field (**Fig. 4C**, *bottom center panel*). Surprisingly, despite its disorganization, the global SMI in adult PA rats was significantly increased (**Fig. 4D**; *U* test p<0.05).

The forward masking paradigm was used to assess fundamental temporal aspects of response suppression. When probes were presented 40 ms after maskers, response suppression was recorded for a broad range of maskers (**Fig. 4C**, *top right panel*). In controls, these areas of suppression were typically V-shaped and centered on and scaled to each neuron's TCs. FMI areas were often very broad, and appeared to extend beyond the tested frequency range. Again, anomalies were detected in PA rats. FMI areas were reduced in size and were often limited to masked domains within TCs' borders. Scattered masker suppressions of probe responses, across intensity-frequency fields, were also recorded. Global measures of FMI were significantly reduced in mature PA rats (**Fig. 4D**; *U* test p<0.05), suggesting an altered capability for processing detailed temporal features of acoustic inputs.

### Reduced high-rate processing capabilities in PA neurons

**Figure 5:**
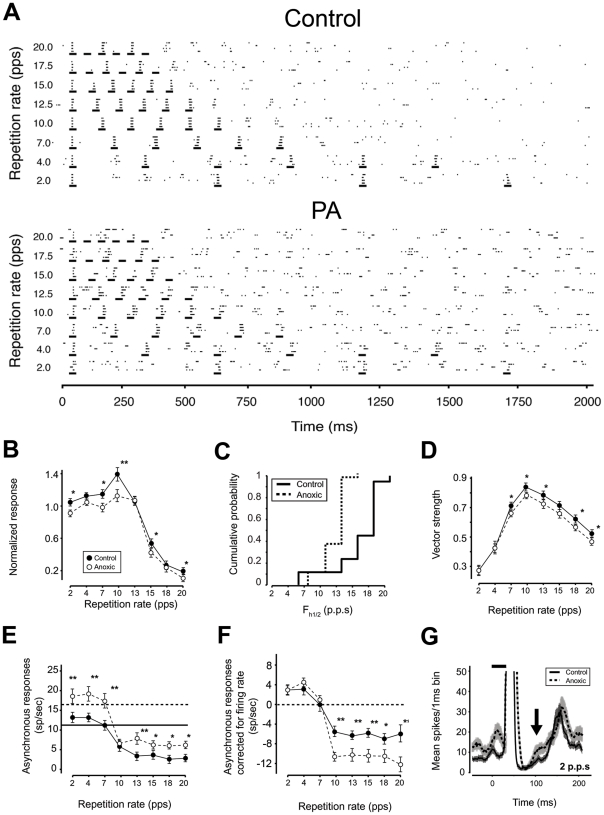
PA affects ability to process high-rate repetitive stimuli. (**A**) Dot raster plots of cortical responses to pulsed noises presented at different repetition rates (ordinate) recorded from a control (upper) and a PA (lower) rats. Most normal (control) A1 neurons could follow repetitive noise bursts up to 12.5 pulse-per-sec (p.p.s.), responding with similar spiking to all six noise-burst events and some neurons could follow rates up to about 20 p.p.s. (e.g., top panel). By contrast, in adult PA rats, most neurons responded in a similar manner to noise burst rates of 10 p.p.s. or lower, but fewer could follow rates of 12.5 p.p.s. and higher (e.g. as in the sample shown in the lower panel). In PA neurons, altered response pattern could be already noted at rates as low as 4 p.p.s.. PBI after spiking-bursts was particularly evident in controls. Total spike numbers recorded after the first noise pulse were not significantly different between the two groups. (**B**) Temporal modulation transfer function of cortical responses from 92 sites in control animals and 76 in PA rats. tMTF in PA rats was significantly reduced at most rates presented. Asterisks mark significantly different values. (**C**) Cumulative frequency histogram showing average highest temporal rates at which cortical responses were half of their maximum (f_h1/2_) for controls (grey) and PA (black) rats. Cumulative distribution showed a significant leftward shift for PA compared to controls rats [*t*-test p<0.001 (1.4766e-14)]. (**D**) Vector strength of cortical responses, a measure of phase-locking to repetitive noise pulses or entrainment. Entrainment was very similar in control and PA rats for low rate repetitive stimuli. For repetition rates of 7 Hz or higher, phase locking capabilities were significantly reduced in PA neurons. (**E–F**) Asynchronous responses to repetitive noise pulses, calculated as the mean firing rate recorded 40 ms after each stimulus onset to the onset of the following stimulus (**E**) and after subtracting the average spontaneous (background) firing rate (**F**). In PA rats significantly larger asynchronous responses paralleled the higher background noise recorded. The level of suppression appeared significantly stronger in PA rats (**F**) because it resulted from the subtraction of a greater spontaneous activity. (**G**) Response dynamics of cortical neurons before and after neurons were activated by repetitive pulsed noise. The arrow shows an average faster return to the baseline in agreement with reduced post-firing suppression in PA rats.

Behaviorally important complex sounds, such as animal vocalizations and human speech, often contain repetitive acoustic sequences. Excitatory-inhibitory alternations are of critical importance for analyzing those sequences [26]. The ability of A1 neurons to respond to repetitive stimuli was assessed by presenting trains of noise bursts at repetition rates ranging from 2 to 20 pulse per second (p.p.s.) at 65 dB (**Fig. 5A**). Temporal modulation-transfer function (tMTF, see methods) showing the average normalized response to the last five noise bursts as function of stimulus repetition rates are shown in **Fig. 5B**. In control rats, most A1 neurons responded with similar spike bursts to all six presentations up to 12.5 p.p.s. (see also [27], [28]). The ability of A1 neurons to respond to all six presentations decreased with increasing the presentation frequencies and fewer neurons responded with the same strength to every noise pulse at 15 and 17.5 p.p.s.. Responses to every presentation at 20 p.p.s. were recorded only from a few cortical sites (**Fig. 5A**, *top panel*).

The average tMTF had poorer responses for higher repetition rates in PA rats than in controls. In adult PA rats, most neurons responded with similar strength to every noise burst at rates of 10 p.p.s. or lower, and a fewer neurons could follow rates of 12.5 p.p.s. and higher (5A *lower panel*). tMTF showed that neurons from adult PA A1 responded with significantly reduced capabilities at most rates presented (**Fig. 5B**). Reduced high-rate processing capabilities were also inferred by the leftward shift in the cumulative distribution of the identified highest temporal rates at which tMTFs were at half their maximum (f_h1/2_) plotted as a function of temporal rates (**Fig. 5C**).

The vector strength (VS) quantifies the temporal fidelity, or entrainment, by which each noise burst evokes time-locked spikes. Rayleigh statistics (RS) estimates VS significance level taking the total number of spikes into account. At 2 and 4 p.p.s. neurons from both groups exhibited a similar degree of entrainment (**Fig. 5D**). However, when bursts rates rose above 7 p.p.s., VS and RS were both significantly reduced in adult PA rats.

Responses to noise bursts occurred mostly between 8 and 45 ms after stimulus burst onset (phase-locking window). Each response window was followed by an epoch of PBI (see also **Fig. 2D**). The average firing rate between phase-locking response windows can be quantified as a function of temporal rates. This ‘asynchronous index’ provides information about neurons' suppression capabilities during repetitive stimulation. Spontaneous firing rate between phase-locking windows were higher in PA rats (**Fig. 5E**) consistent with reduced inhibitory suppression in the asynchronous epochs (**Fig. 5E** and **5G**, but see also **Fig. 2D**). At 2 and 4 p.p.s., asynchronous responses were greater than the average spontaneous firing level in both groups; they were equal to their respective spontaneous firing at 7 p.p.s., but fell below that level for rates between 10 and 20 p.p.s.. After spontaneous firing rates were subtracted, asynchronous responses were similar in both groups at low temporal rates (**Fig. 5F**), but significantly different at 10 p.p.s. and higher rates. The return to the baseline was faster in PA neurons, consistent with reduced levels of inhibition (**Fig. 5G**). Altogether these data suggests that degraded inhibitory mechanisms may underlie reduced high-rate processing capabilities recorded in adult rats exposed to PA. PA reduces A1 neuron potentials to follow high rate repetitive stimuli, without affecting the capabilities to follow low rates.

### Peripheral cochlear sensory epithelium was not affected by PA

**Figure 6:**
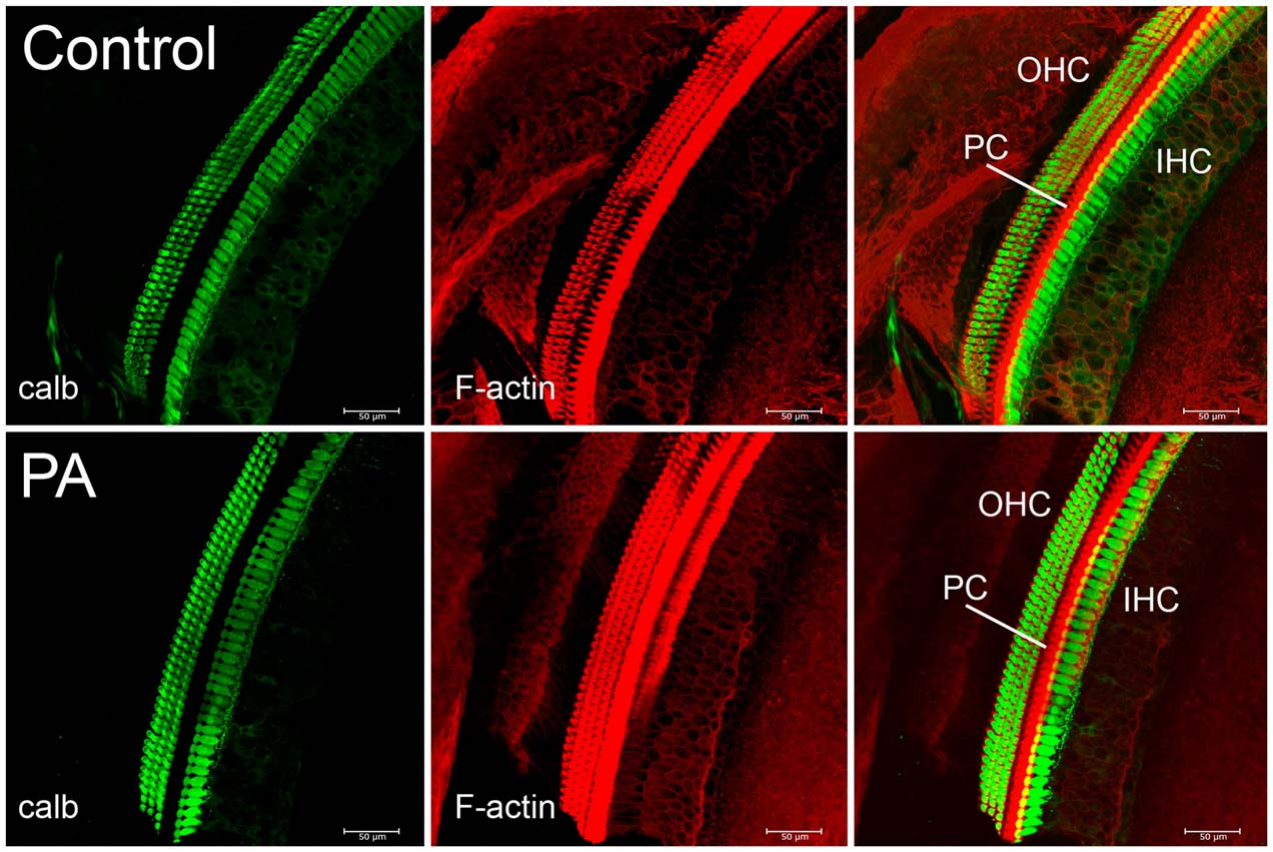
Immunostaining of the hair cells of the basal turn of the organ of Corti. *Left panels*. Green indicates calbindin staining of three rows of outer hair cells (OHC) and one row of inner hair cells (IHC). Calbindin was selected in order to assess cellular loss in the cochlear sensory epithelium in rats exposed to PA. Calbindin immunostaining revealed no difference in the number of either inner or outer hair cells between control and experimental cochleae. *Center panels*. Red indicates TRIC-phalloidin staining of the F-actin filament expressed by OHC, IHC and pillar cells, a variety of support cells of the organ of Corti. F-actin expression was similar in control and PA rats suggesting the integrity of the stereocilia of the cochlear epithelium. *Right panels*. Merged images with FITC-green and TRITC phalloidin-red. Scale bars correspond to 50 µm.

Although these deficits have been recorded in the primary auditory cortex of adult rats, this PA-induced damage could have occurred at any or all levels of the auditory system, beginning with the inner ear itself. The cochlear sensory epithelium was therefore examined in 2 controls and 3 PA adult rats. The organ of Corti were extracted from decalcified cochleae, processed, immunolabeled and compared. As shown in **Fig. 6**, calbindin and F-actin immunostaining revealed three rows of outer hair cells (OHC) and a row of inner hair cells (IHC) in both control (top panels) and PA rats (bottom panels). F-actin immunostaining revealed also pillar cell bodies (center panels). Confocal images of the calbindin and F-actin double labeling showed that the organs of Corti of adult PA rats were structurally similar to those of controls. Altogether these data suggest that the recorded deficits in adult PA rats were not caused by damage of the peripheral cochlear sensory epithelium or other gross structural alterations of the inner ear, and may therefore be of central origin.

## Discussion

Reduced response amplitudes, increased onset latencies, prolonged response durations selective for higher intensity sounds, reduced post-burst inhibition, broader and less regular tuning curves, stronger but less organized simultaneous masking, weaker and altered forward masking, degraded temporal processing representations of repetitive sounds, associated with the integrity of the peripheral cochlear sensory epithelium were all documented in adult rats' A1 neurons exposed to experimental PA.

### Altered excitatory-inhibitory balance in PA rats

Most A1 neurons respond to tones with brief onset bursts followed by prolonged inhibitions [20], [26], [29], [30]. For most of A1 neurons, evoked responses were simple functions of stimulus intensity: excitatory and inhibitory response amplitudes decreased and onset latencies increased monotonically with decreasing sound intensities [19], [20]. In adult PA rats, combined excitatory and especially inhibitory responses were reduced in amplitude, and onset latencies were delayed. Response burst durations in controls grew with increasing intensity and then became shorter again at higher levels, presumably because of growing PBI (see Scholl and Wehr 2008). By contrast, in PA neurons, responses to higher level sounds were more sustained than in controls, and bursts were longer than at lower levels because post-burst inhibition was weaker.

Response amplitudes, onset delays and durations recorded in adult PA rats approximately corresponded to those recorded in controls for sound intensities that were 20–30 dB lower. This disparity matched behavioral threshold differences reported in an earlier study, thus providing electrophysiological evidence for the origin of that observed performance deficit [16]. On the other hand, other response differences that distinguished neuronal response selectivity in time and frequency, modulation response characteristics, or forward, simultaneous or backward masking had no such intensity-shifted equivalence in PA and normal rats.

### Degraded temporal representation of sounds in PA neurons

Abnormal cortical excitation and inhibition were evident in degraded time slice analyzed TCs. In normal A1 neurons, Schreiner et al. [21] have shown that responsiveness is expressed by a gradual progression of activity across the intensity-frequency fields, in time. Neurons first respond to a broad frequency range of high intensity tones, displaying low spectral selectivity, and then in subsequent time-slices to a narrower range of frequencies at lower intensities, around neurons' CF. By acting as independent variables, response onset latencies and durations, contribute to the transient expression of evoked activity across intensity-frequency fields as a function of stimulus intensity. This enables neuronal ensembles in the tonotopic auditory cortex to represent with high precision relevant complex segments in sound streams, i.e. syllables or words [21]. In PA neurons, activity evoked by the broad frequency range of high intensity stimuli persisted and overlapped with responses to the narrow frequency range of near-CF tones. This degraded responsiveness effectively influences spectrally selective activity evoked by near-CF stimuli, and thereby presumably affects the ability of A1 neurons to represent complex sound features with similar precision.

### Altered simultaneous and forward masking in PA neurons

Simultaneous masking was, overall, surprisingly stronger in PA rats. This pattern of suppression was similar to that recorded in the immature auditory cortex [23], but it differs from it because a stronger SMI in adult PA rats correlated with increased response suppression randomly distributed across the intensity-frequency spectrum. In agreement with its suggested role in shaping TCs spectral selectivity [25], increased SMI was associated with substantially degraded spectral feature representation by A1 neurons [16]. Reduced sideband inhibitory areas may account for increased TC bandwidths. Increased SMI could also contribute to delayed response onsets, reduced excitatory response amplitudes, and associated PBI. Altogether these data suggest a complex “permissive-integrative” role on neuronal responsiveness in order to shape neurons spectral and temporal features. SMI has been argued to be essential to A1 neurons' abilities to provide an estimation of complex spectral analysis and prediction of frequency-modulated sweep and “ripple spectra” [31], [32], [33]. SMI is mostly cortical in origin and it is sensitive to the GABA_A_-receptors blocker bicuculline [23]. GABA_A_-receptor mediated currents, because of reversed chloride ion concentrations at the dendritic level, have a permissive role on evoked responses [34]. The striking difference in SMI areas associated to TCs similar in shapes, BWs and CFs are consistent with the suggested complex integrative role rather than solely shaping TCs (see also [25]).

Consistent also with a diminished post-burst inhibition, reduced suppression capabilities in A1 cortical neurons of adult PA rats likely contributed to a decreased FMI. FMI areas normally decrease in size with increasing masker-to-probe onset intervals [22], [24]. Delayed response onsets and shorter response durations (particularly to low-level tones) recorded in PA neurons may contribute to reduced FMI areas. FMIs have been reported at several levels of the auditory system [35], [36]) and their feed-forward effects can largely account for FMI recorded at the cortical level [25]. However, others have argued that FMI is at least partly cortical in origin [24]. FMI is long-lasting and bicuculline-insensitive consistent with a GABA_B_-mediated inhibition [37]. It has been suggested that FMI could operate as a temporal filter creating a temporal feature detection mechanism [24].

### Reduced high-rate processing capabilities in PA neurons

Reduced FMI underlies the reduced neuronal capabilities for processing repetitive sequences in adult PA rats. Repetitive noise pulses evoked responses characterized by short bursts of spikes followed by silent intervals. This excitation-inhibition sequence has been suggested to underlie precise response timing and determine response-limiting rates for a specific neuron. Responses to noise bursts are not based on spectral features, and following-rate limitations are a function of the silent periods between stimuli more than stimulus periodicity, thus providing additional evidence that the functionality of inhibitory networks has been impaired in PA rats (see also [26]). The tMTF, expressing neural discharge rate as a function of the stimulus repetition rate, reflects the succession of excitatory and inhibitory cortical events in temporally modulated or repetitive sounds [26], [27]. tMTF measures locked response magnitude to repetitive stimuli, whereas VS measures the degree of entrainment (providing complementary information on cortical temporal processing). In PA rats, tMTF amplitudes were reduced at the majority of the presented rates. VSs at 2 and 4 p.p.s. were similar in both groups, suggesting that PA did not affect lowest rate processing. As repetition rates increased phase-locking capabilities of A1 neurons were strongly reduced. Preserved low repetition rate processing and response bursting capabilities in the cortex of PA rats may have relevant implications in terms of reorganizational plasticity. This temporal following capability has been shown to be cortical in origin. Monkeys trained to discriminate between two different noise burst rates loose this capability after ‘extended’ bilateral ablation of their primary auditory cortex [38]. Enduring impairments in processing repetitive stimuli have also been demonstrated by rearing rats in pulsed or notched noises [28], [39]. By contrast, rats trained in an operant behavioral task with high-rate repetitive stimuli showed improved repetitive stimuli processing capabilities [27]. Finally, in line with reduced levels of inhibitions, baseline spontaneous firing rates and asynchronous responses (anti-phase for bursts) were greater in PA rat A1 neurons than in controls. When baseline firing was subtracted from asynchronous firing, PA neurons showed a greater level of asynchronous response suppression. Altogether these data suggest that either a strong tonic inhibition and/or weaker cortical inputs may fail to engage the cortex via what are normally balanced excitatory-inhibitory dynamics. Cortical inhibition could be present but not working effectively to help produce coordinated spiking. In such a scenario, neurons fire more randomly, and are less available at any given time to respond to repetitive noise pulses.

### Auditory processing deficits are central in origin

In summary, in the present rodent model of PA, brief acute episodes of total asphyxia, which have little or no effects on motor [40] and somatosensory [41] systems, can generate prominent damage to the auditory system [16] as shown by abnormal cortical neuronal representations of spectral and temporal features of complex sounds. Although recorded in the primary auditory cortex, this enduring PA-induced damage could have occurred at any or all levels of the auditory pathway, beginning with the inner ear itself. However, in this model of experimental PA the cochlear sensory epithelium was not damaged, and both inner and outer hair cells number and morphology were not altered. This is in line with a previous study using the same experimental PA model [16], in which ABR's peak I (acoustic nerve) and peak II (cochlear nucleus) were not significantly different from controls. Altogether these data suggest that the degraded auditory processing recorded in A1 may start at the level of the brain stem auditory nuclei.

Highly metabolically active brain stem auditory nuclei are very sensitive to PA asphyxia [8], [42], [43], [44]. These nuclei process and relay multiple sound features including salient timing and intensity cues of communication calls (cochlear nucleus; [45]), precise timing cues for sound localization (superior olivary complex; [46]), and detection of sound onset with a damping function (inferior colliculus; [47]). Degraded timing-intensity sound features relayed by brain stem auditory nuclei to the primary auditory cortex may account for part of the deficits recorded in PA rats. The precision with which these timing-intensity cues are processed and relayed depends strictly on the finely-tuned excitatory-inhibitory balance within the auditory system [46]. A disruption in this excitatory-inhibitory balance, may underlie the timing-intensity precision deficits and the increased behavioural and hearing thresholds recorded in A1 of adult PA rats [16] or other behavioural and electrophysiological abnormalities described in monkeys after hasty umbilical cord clamping [8], [13].

### Potential relations with auditory perceptual deficits in neurodevelopmental disorders

A disruption in the excitatory-inhibitory balance has been suggested to play a critical role in the pathogenesis of autism [48]. Reduced inhibition may account for high prevalence of autism and epilepsy co-morbidity (up to over 60% depending on the study [49]); whereas reduced inhibition in one system or reduced inhibitory influence from another may favour the development of savants. Moreover, structural alterations of BS auditory nuclei, increased hearing threshold and orientation to sounds or modality specific impairments and cortical evoked potentials anomalies recorded in PA monkeys after *in utero* (immediate) clamping of the umbilical cord [8], [9], [42] have also been described in autistic infants [7], [17], [50]. Indeed, hearing deficits including abnormal (delayed) auditory brainstem responses (ABRs; [17]), temporal processing deficits [51], reduced discrimination and orientation to speech [14], increased hearing thresholds [15], and increased perception of loudness (i.e. hyperacusis; [52]) have been observed in the large majority of autistic individuals. This variety of perceptual communication dysfunctions may underlie the multifaceted characteristics of the autistic spectrum disorder. In conclusion, even if auditory system's anoxic injuries may not be causal in autism, they might play a role in a consistent number of cases, especially considering the role of auditory perception and production in the communication domain.

## Materials and Methods

### Ethics Statement

All experimental procedures described in this study were approved by the National Institutes of Health, and the Committee on Animal Research at the University of California, San Francisco, authorization protocol #AN077742-02D, and described in details in an earlier report [40].

### Experimental procedure

Briefly, twenty-four newborn rats from four litters were randomly assigned to control or experimental groups. Pups assigned to the experimental group experienced an epoch of anoxia on the day of birth (P0), and again on the following day (P1). Pups were placed over a thermal blanket (37°C) in a Plexiglas, airtight chamber and pure nitrogen gas (100% N_2_) was passed through the inlet. After about 1 min, the pups became hyperactive, and within the next 1–2 min, hyperactivity was followed by a change in skin color from light pink to bluish, and by movement cessation and sporadic gasping. Twelve minutes after the beginning of the hyperactivity, the N_2_ was turned off, the chamber was opened, and pups quickly removed, resuscitated, and left in normal atmospheric conditions until they returned to their original pink color and were breathing regularly. The procedure was repeated 24 h later. Atmospheric air was used in control rats [41].

### Electrophysiology

Most responses were recorded via a dense, uniform sample spanning the primary auditory cortex (A1) in fifteen (six control and nine PA animals) young adult rats ranging between 90 and 180 days of age. Animals were anesthetized with 60 mg/kg pentobarbital i.p. and maintained at an areflexic level of anesthesia throughout the recording experiment. Tone pips (25-ms durations with 3-ms on and off ramps) delivered at 2 pulses per second with a calibrated sound delivery system (Tucker–Davis Technologies, Gainesville, FL) were used to examine response properties of A1 neurons. Selective frequency-intensity responses were documented by recording extracellular single or multiple units at 121 sites in controls and at 400 sites in PA animals. All recordings were with parylene-insulated tungsten microelectrodes (1-µm tip diameter, 1–2 MΩ at 1 kHz, FHC, Bowdoinham, ME). Sampled units were in the middle cortical layers (layers IV-V) in the right hemisphere. Tone pips were randomly presented at 50 different frequencies and 8 different intensity levels to derive tuning curves for every neuron (cortical site) sample. Characteristic frequencies (CFs) were defined at each site as the frequency that evoked reliable spike discharges at the lowest stimulus intensity. Tuning curves (TCs), were defined as V-shaped continuous areas of responses to a finely graded (1/8^th^ octave) series of test tones (tone pips) presented at graded intensity steps (10 dB SPL). Thresholds were defined as the lowest intensity at which reliable responses were recorded. MAP software [53] was used to mark cortical sites on a digitized image of the cortex obtained with a CCD camera. Custom analysis programs were used to reconstruct frequency regions from the pattern of electrode penetrations marked on the cortical surface. Boundaries of A1 were defined by stepwise changes in response latencies and by reversal in tonotopic order, as in earlier studies.

Data are presented as mean±s.e.m. Mixed ANOVA was applied to test for the significance of differences within and between groups. Student *t*-test and the nonparametric Mann–Whitney *U* test were used in all other cases or as a supplemental statistical analysis. Values of *p*<0.05 were considered significant.

Inhibitory receptive fields were derived at 35 controls and 42 PA sites using two-tone stimulus paradigms in which varying “masker” tones suppressed cortical responses to a fixed “probe” tone [23]. The probe tone was 10–20 dB above the unit threshold, at the units' best frequency. A total of 400 maskers were presented in random order. The range of maskers completely spanned and extended beyond the limits of the frequency-intensity field of each studied unit. The two-tones were either presented simultaneously (simultaneous masking inhibition, SMI) or with an onset asynchrony of 40 ms (forward masking inhibition, FMI).

Temporal modulation transfer functions (tMTFs) were obtained by presenting trains of six noise bursts (25 ms duration with 5 ms ramps) at 65 dB SPL. Each sequence was delivered four times, at each of 8 repetition rates (2–20 pulses per second), all in random order. Normalized cortical responses at each repetition rate were calculated by dividing the average response magnitude to the last five noise pulses by the response magnitude to the first pulse. tMTFs plotted the normalized cortical response as a function of temporal rate [28]. Highest temporal rates at which the tMTF was at least half of its maximum, referred as f_h1/2_, provided a simple measure of cortical efficiency for processing high-rate stimuli. Vector strength (VS) and Rayleigh statistics (RS) were used to quantify cortical temporal fidelity (see [27]).

### Immunohistochemistry

Cochleae were removed from the temporal bone of 5 rats (2 controls and 3 experimental) after transcardial perfusion with 4% paraformaldehyde in 0.2 M phosphate buffer (pH 7.4). Cochleae were also perfused through the *finestra ovalis* and post-fixed in paraformaldehyde 4% for 24 hours. Cochleae were then incubated in 0.5 M EDTA solution for 10 days for the decalcification process. After rinsing (2×30 min in PBS) they were embedded in 3% agarose (Biorad, Segrate (MI), Italy) and cut into 250-µm sections in phosphate-buffered saline (PBS) by using a vibratome (Leica VP1000S, Wetzlar, Germany). For immunohistochemistry cochlear sections were pre-incubated in a blocking solution (1%Triton X-100/2%BSA/1XPBS) for 3 hours and incubated at 4°C overnight in the primary antibody (1∶1500 polyclonal anti-calbindin 28 K; Swant, Bellinzona, Switzerland). The following day sections were washed with PBS/BSA and then incubated in secondary fluorescent antibody AlexaFluor 488 (1∶500 in PBS/BSA; Vector Laboratories, Burlingame, CA). Tetramethyl rhodamin isothiocyanate-conjugated phalloidin (1∶500 in PBS/BSA; Molecular Probes) was also used to visualize filamentous actin. Mowiol 10% (Calbiochem, La Jolla, CA) was used to mount the specimen and cover-slipped slides were analyzed by Carl Zeiss laser scanning System confocal microscope.
