# Correction: Perinatal Asphyxia Affects Rat Auditory Processing: Implications for Auditory Perceptual Impairments in Neurodevelopmental Disorders

**DOI:** 10.1371/annotation/56c65e4d-1fce-4378-a5a2-97f799b6e827

**Published:** 2011-01-11

**Authors:** Fabrizio Strata, Ivilin P. Stoianov, Etienne de Villers-Sidani, Ben Bonham, Tiziana Martone, Tal Kenet, Edward F. Chang, Vincenzo Vincenti, Michael M. Merzenich

There was an error in funding information regarding the year the PRIN grant was granted to FS. The correct financial disclosure should be: "This work was supported by the National Institutes of Health Conte Center grant MH77970 (M.M.M.), the John C. and Edward Coleman Memorial Fund (M.M.M.), and the Italian Ministry of University and Research, PRIN 2008 (F.S.). The funders had no role in study design, data collection and analysis, decision to publish, or preparation of the manuscript."

